# Mechanisms of the Testis Toxicity Induced by Chronic Exposure to Mequindox

**DOI:** 10.3389/fphar.2017.00679

**Published:** 2017-09-26

**Authors:** Qianying Liu, Zhixin Lei, Anxiong Huang, Qirong Lu, Xu Wang, Saeed Ahmed, Ihsan Awais, Zonghui Yuan

**Affiliations:** ^1^National Reference Laboratory of Veterinary Drug Residues (HZAU) and MAO Key Laboratory for Detection of Veterinary Drug Residues, Wuhan, China; ^2^MOA Laboratory for Risk Assessment of Quality and Safety of Livestock and Poultry Products, Huazhong Agricultural University, Wuhan, China; ^3^Hubei Collaborative Innovation Center for Animal Nutrition and Feed Safety, Wuhan, China; ^4^Department of Biosciences, COMSATS Institute of Information Technology, Sahiwal, Pakistan

**Keywords:** mequindox, oxidative stress, reproductive toxicity, blood-testis barrier, metabolites

## Abstract

Mequindox (MEQ) is a synthetic antimicrobial agent widely used in China since the 1980s. Although the toxicity of MEQ is well recognized, its testis toxicity has not been adequately investigated. In the present study, we provide evidence that MEQ triggers oxidative stress, mitochondrion dysfunction and spermatogenesis deficiency in mice after exposure to MEQ (0, 25, 55, and 110 mg/kg in the diet) for up to 18 months. The genotoxicity and adrenal toxicity may contribute to sperm abnormalities caused by MEQ. Moreover, using LC/MS-IT-TOF analysis, two metabolites, 3-methyl-2-(1-hydroxyethyl) quinoxaline-*N*4-monoxide (M4) and 3-methyl-2-(1-hydroxyethyl) quinoxaline-*N*1-monoxide (M8), were detected in the serum of mice, which directly confirms the relationship between the *N*→O group reduction metabolism of MEQ and oxidative stress. Interestingly, only M4 was detected in the testes, suggesting that the higher reproductive toxicity of M4 than M8 might be due to the increased stability of M4-radical (M4-R) compared to M8-radical (M8-R). Furthermore, the expression of the blood-testis barrier (BTB)-associated junctions such as tight junctions, gap junctions and basal ectoplasmic specializations were also examined. The present study demonstrated for the first time the role of the M4 in testis toxicity, and illustrated that the oxidative stress, mitochondrion dysfunction and interference in spermatogenesis, as well as the altered expression of BTB related junctions, were involved in the reproductive toxicity mediated by MEQ *in vivo*.

## Introduction

**FIGURE 1:**
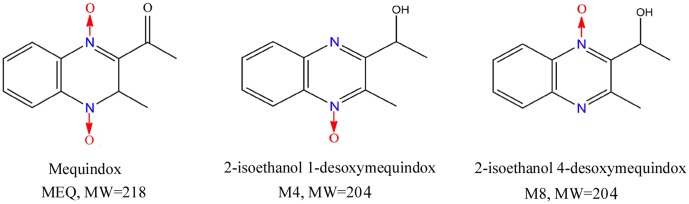
The chemical structures of mequindox (MEQ), 2-isoethanol1-desoxymequindox (M4) and 2-isoethanol 4-desoxymequindox (M8).

Mequindox (3-methyl-2-acetyl-*N*-1,4-dioxyquinoxaline, C_11_H_10_N_2_O_3_; MEQ) (**Figure 1**) is structurally similar to QdNOs, a group of chemicals consisting of one or two acyclic chain moieties combined with a quinoxaline ring. QdNOs are synthetic agents with a wide range of biological properties including antibacterial, anti-candida, anti-tubercular, anti-cancer, anti-protozoal and growth-promoting activities (Wu et al., 2007; Vicente et al., 2009; Wang et al., 2011a, 2015, 2016d; Cheng et al., 2015; Liu Q. et al., 2016). Four QdNO derivatives, CBX, OLA, QCT and cyadox (CYA), have been developed for use in livestock and poultry farming, contributing to their significant antibacterial abilities and growth-promotion properties (Wang et al., 2015; Zhang et al., 2015). However, CBX and OLA have been banned in food-producing animals by European Commission since 1998 because of their potential genotoxic and carcinogenic toxicity (Wang et al., 2016d). MEQ is a relatively new synthetic antibacterial agent that was widely applied in China in pigs and chickens owing to its strong inhibitory effect against both gram-positive and -negative bacteria (Ihsan et al., 2010, 2011, 2013; Huang et al., 2015).

A large amount of evidence has suggested that the *N*→O group reduction is a major concerned metabolic pathway of QdNOs (Cheng et al., 2015, 2016; Liu Q. et al., 2016); accompanying this, some ROS and radical intermediates have emerged that ultimately cause oxidative stress (Liu et al., 2017). It was revealed that oxidative stress plays a critical role in the damage caused by QdNOs such as apoptosis, DNA and lipid damage *in vitro* and *in vivo* (Chowdhury et al., 2004; Azqueta et al., 2007; Yang et al., 2013; Zhang et al., 2014; Wang et al., 2015; Liu et al., 2017). The genotoxicity induced by QCT, OLA and MEQ was found to be closely related to oxidative stress (Zou et al., 2009; Huang et al., 2010; Liu W.Y. et al., 2012; Dai et al., 2015; Yang et al., 2015; Wang et al., 2016d). In a recent study to investigate the mechanism of genotoxicity induced by QCT, the production of ROS during the metabolism of QCT by xanthine oxidoreductase (XOR) was considered a main factor in DNA strand breakage (Wang et al., 2016d). Thus, it was suspected that oxidative stress may be involved in the reproductive toxicity in mice after chronic exposure to MEQ.

Importantly, apart from oxidative stress, the *N*→O group reduction metabolites of QdNOs were also found to be associated with their toxicity (Hao et al., 2006; Chen et al., 2008, 2009; Zhang et al., 2012, 2015; Wang et al., 2015; Liu Q. et al., 2016; Liu et al., 2017). The primary metabolites of MEQ, *N*1-MEQ and bidesoxy-mequindox, exhibited genotoxicity in *in vitro* and *in vivo* short-term tests (Liu Q. et al., 2016). We recently showed that 2-isoethanol 1-desoxymequindox (M4) and 2-isoethanol 4-desoxymequindox (M8) (**Figure 1**) were involved in liver toxicity in mice after exposure to MEQ for 11 months (Liu et al., 2017). It was revealed that oxidative stress and metabolites of MEQ were regarded as important toxicity mechanisms in *in vivo* models of the adrenal gland (Huang et al., 2009), liver (Wang et al., 2011b; Liu et al., 2017), spleen (Wang et al., 2011b), and endocrine and reproductive system (Ihsan et al., 2011). The *in vitro* adrenal toxicity study on H295R cells that originated from a human adrenocortical carcinoma has further confirmed this conclusion (Wang et al., 2016c). Therefore, the metabolites and oxidative stress were thought to participate in reproductive toxicity caused by MEQ. However, to date, the mechanism underlying the correlations of these two factors in the reproductive toxicity induced by MEQ *in vivo* still remains unclear.

A previous *in vitro* study reported that MEQ could be metabolized into 10 metabolites after incubation with liver microsomes of pigs, chickens, and rats (Liu Z.Y. et al., 2010). Among these metabolites, M4 was identified as a common and major metabolite of MEQ *in vivo* by quantitative analysis using isotopic tracing (Huang et al., 2015). A total of five metabolites without intact MEQ had been identified in chicken plasma samples by high performance liquid chromatography combined with ion trap-time of flight-mass spectrometry (Liu Y.C. et al., 2010). Additionally, MEQ and its six metabolites were detected in plasma samples of rats after a single i.v. and p.o. administration of MEQ at the dose of 10 mg/kg b.w. (Li et al., 2012). Recently, in a study to investigate the metabolism, distribution, and elimination of MEQ *in vivo*, intact MEQ was present in both urine and feces in rats, in urine at a comparable low level in pigs, and no intact MEQ was observed in the excreta and tissues in chickens (Huang et al., 2015). MEQ was metabolized extensively in animals with a generation of 13 metabolites and most of the metabolites were found consistently in pigs, rats, and chickens, demonstrating that the main metabolism route are similar in different species (Huang et al., 2015). Regarding the reproductive toxicity induced by MEQ, only one study has been conducted and demonstrated that the chronic exposure of MEQ triggered reproductive toxicity in male rats (Ihsan et al., 2011), suggesting a certain risk of chronic MEQ exposure on the fertility of animals and human. MEQ is widely used in animal production in China for many years (Liu J. et al., 2012; Wu et al., 2012; Li et al., 2014; Wang et al., 2016b), however, its reproductive toxicity has not been adequately investigated. To our knowledge, a previous study revealed that 2-isoethanol 4-desoxymequindox (M11), a metabolite of MEQ, was detected in the testis of wistar rats (Ihsan et al., 2011), suggesting that MEQ may disrupt the BTB in the testes *in vivo*. Therefore, the objective of this study was to clarify the reproductive toxicity of MEQ *in vivo*, and to further elucidate the role of BTB-associated junctions and the potential toxic metabolites of MEQ in the testis of male mice, which will improve the prudent use of MEQ for public health.

Based on the above information, we hypothesized that the MEQ may induce reproductive toxicity in male mice. The metabolites of MEQ and oxidative stress involved in the metabolism of MEQ may disrupt the BTB in testes of mice. In the present study, we comprehensively evaluated the reproductive toxicity in Kunming mouse after exposure to MEQ for 18 months. To verify the above hypothesis, we investigated (1) the effect of MEQ on body and testis weight, and histological structures in testes, (2) the effect of MEQ on ultrastructural changes in testes by TEM observations, (3) the activity of MDA and 8-OHdG in serum, (4) the identification of metabolites of MEQ in the serum and testis by LC/MS-IT-TOF analysis, and (5) whether MEQ changed the mRNA expression of BTB-associated genes including basal ESs (*N*-cadherin, α-catenin and β-catenin), GJs (Connexin-43), and TJs (CAR, F11R, Occludin and ZO-1) in the testes of mice.

## Materials and Methods

### Chemical Reagents

Mequindox (C_11_H_10_N_2_O_3_, molecular weight 218.21 g/mol, CAS No: 60875-16-3, purity 98%) was obtained from Beijing Zhongnongfa Pharmaceutical Co., Ltd. (Huanggang, China). Bouins’ solution, glutaraldehyde, cacodylate, acetonitrile, formic acid, trichloroacetic acid and dichloromethane were purchased from Sigma (St. Louis, MO, United States).

### Test Animals

Forty specified pathogen-free (SPF) Kunming male mice (6- to 7-weeks-old, 30 ± 5 g) were purchased from the Center of Laboratory Animals of Hubei Province (Wuhan, China). The individual body weight (BW) of each mouse was within ±20% of the average. The mice were maintained in a room conditioned at 22 ± 3°C, a relative humidity of 50% ± 20%, and a 12 h light/dark cycle. The mice were housed five per group per sex in shoebox cages with hardwood shavings as bedding. The shoebox consisted of polypropylene PP plastics, and the box lid is stainless steel with many fences. The box size is 290 mm × 180 mm × 160 mm. Food and tap water were supplied *ad libitum.* The diet were purchased from the Center of Laboratory Animals of Hubei Province (Wuhan, China). Prior to use, the tap water and bedding material were autoclaved by high temperature sterilizing oven, and the diet was sterilized by ultraviolet light. All animal work was in compliance with the NIH Publication “The Development of Science Based Guidelines for Laboratory Animal Care” (NRC, 2004). The experimental procedures involving mice were performed in accordance with the guidelines of the Committee on the Care and Use of Laboratory Animals of China (permit SYXK 20070044). In the present study, the mice were handled in accordance with the protocols approved by the Ethical Committee of the Faculty of Veterinary Medicine (Huazhong Agricultural University).

### Experimental Design

According to the Organization for Economic Cooperation and Development (OECD) Guideline 453 and Procedures for toxicological assessment of food in China, the high-dose level should cause some toxic effect performance or damage, and the low-dose group may not show any toxic effects, but should be 1–3 times greater than the clinical dose (GB15193.17, 2003; OECD, 2009). In a previous sub-chronic and chronic toxicity study, MEQ in 110 mg/kg diet made an increase in plasma potassium (K^+^) level without growth inhibition, and this dose was determined to be no-observed-adverse-effect level (Ihsan, 2011). Therefore, the 110 mg/kg diet was selected as the high dose, and 55 mg/kg for the middle and 25 mg/kg for the low dose, respectively.

Prior to the initiation of dosing, mice were evaluated for any signs of disease and weight gain by being quarantined for 1 week. After acclimatization for 1 week, the male Kunming mice were randomly divided into four groups (*n* = 10), including a control group and three MEQ treated groups. The control group received the basic diet without feed additives. Three MEQ treated groups were administered the same diet supplemented with 25, 55, and 110 mg/kg MEQ, respectively. The treatment period lasted for 18 months. Symptoms and/or mortality were observed and carefully recorded each day during the 18 month period.

### Preparation of Testes

Following 18 months of MEQ administration, the mice were weighed and sacrificed after being anesthetized. After weighing the body and testes, the relative of testis weight was calculated as the ratio of testes (wet weight, mg) to BW (g). The testes were excised, rinsed in phosphate buffered saline (PBS), and quickly frozen at -70°C.

### Histopathological Examination

The histopathological tests were performed using standard laboratory procedures. The right testis from the males (*n* = 5) were immediately fixed in Bouins’ solution for 24 h. After fixation, the testes were rinsed by running water and embedded in paraffin blocks, then sliced into 5 μm thick sections and placed onto glass slides with hematoxylin-eosin (HE) staining. Finally, the morphological alterations of testes were observed under an optical microscope (Olympus BX 41, Japan).

### Transmission Electron Microscope (TEM) Observation

The right testis from other males (*n* = 5) fixed with 2.5% glutaraldehyde in 0.1 mol/dm^3^ cacodylate buffer for 4 h, then followed by washing three times with 0.1 mol dm cacodylate buffer (pH 7.2–7.4) and being placed in 1% osmium tetraoxide for 1 h. A graded series of ethanol (75, 85, 95, and 100%) was used to dehydrate the specimens and they were then embedded in Epon 812. Ultra-thin sections (70 nm) were contrasted with lead citrate for 10 min and uranyl acetate for 30 min, before being observed with an H-7650 TEM (Hitachi, Japan).

### Oxidative Stress Assay

Malondialdehyde and 8-OHdG kits were obtained from Nanjing Jiancheng Bioengineering Institute (Nanjing, China). The effects of MEQ on the activity of 8-OHdG and MDA in the serum of male mice were examined. An assay of MDA level was performed using a commercial kit. 8-OHdG was assayed using commercial ELISA kits. The data were analyzed according to the manufacturer’s instructions. The protein concentration in testes was determined using the BCA protein assay kit as standard.

### LC/MS-IT-TOF Analysis of MEQ and Its Metabolites in Serum and Testis

The MEQ and its metabolites in the serum and testes were detected by the hybrid IT/TOF mass spectrometry coupled with a high-performance liquid chromatography system (Shimadzu Corp., Kyoto, Japan). The liquid chromatography system (Shimadzu) was connected with a DGU-20A3 degasser, a photodiode array detector (SPD-M20A), a solvent delivery pump (LC-20AD), an autosampler (SIL-20AC), a communication base module (CBM-20A) and a column oven (CTO-20AC).

#### Selection of Pretreatment Methods of Samples

In this study, the pretreatment methods of serum and testis were according to the previous studies (Liu Z.Y. et al., 2010; Ihsan et al., 2011). In preliminary experiment, a total of 1.0 ppm MEQ standard was added into 200 μL of the serum and 0.1 g of the testis from the control group, respectively. After pretreatment using the same methods as described detailedly below, the mixture (200 μL) of serum and testis were prepared for LC/MS-IT-TOF analysis, respectively.

#### LC/MS-IT-TOF Analysis of MEQ and Its Metabolites in Serum

Here, 200 μL of the serum sample was mixed with 600 μL extraction reagent of acetonitrile and vortex-mixed for 5 min. Then, the solution was centrifuged at 10,000 × *g* for 15 min in a model omni mixer homogenizer 17106 (OMNI International, Waterbury, CT, United States) to collect the supernatant. The residual sediment was applied to extract MEQ and its metabolites again following the above steps. The supernatant from the two extractions was merged and dried using N_2_ at 40°C water baths. After drying, the residue was dissolved with 200 μL solution of LC-MS/MS mobile phase [acetonitrile: 0.1% formic acid (1:9, v/v)] to prepare for LC/MS-IT-TOF analysis.

#### LC/MS-IT-TOF Analysis of MEQ and Its Metabolites in Testis

Next, 0.1 g of the testis sample from the left testis from all males (*n* = 10) was homogenized with 4.5 mL of 40°C distilled water at the speed of 10,000 × *g* for 3 min in a model omni mixer homogenizer 17106 (OMNI International, Waterbury, CT, United States). Then 0.5 mL trichloroacetic acid was added. After vigorous shaking, the homogenate was centrifuged at 10,000 × *g* for 15 min to collect the supernatant. The mixed reagent [dichloromethane: acetonitrile (2:1, v/v)] was used to extract MEQ and its metabolites twice. Then, 3.0 mL mixed reagent was added to the supernatant and vortex-mixed for 5 min. After vigorous shaking, the solution was centrifuged at 10,000 × *g* for 15 min to obtain the lower liquid. Then, the lower liquid from the two extractions was merged and dried using N_2_ in a 40°C water bath. The residue was reconstituted in 5 mL 5% methanol. The reconstitution fluid was applied to the methanol (3.0 mL) and water (3.0 mL) pre-washed HLB 3cc cartridge (Waters Corporation, Milford, MA, United States). The reconstitution fluid was then sequentially washed with 3.0 mL 5% methanol in water and 5 mL methanol. The extracts of testis were eluted into plastic tubes and evaporated to dryness under N_2_ at 40°C water baths. After drying, the residue was dissolved with 500 μL solution of LC-MS/MS mobile phase [acetonitrile: 0.1% formic acid (1:9, v/v)] and passed through a 0.22 μm filter membrane. The mixture (200 μL) was prepared for LC/MS-IT-TOF analysis.

#### Chromatographic Conditions

Separation of MEQ and its metabolites in the serum and testis was performed on a VPODS column (150 mm × 2.0 mm; particle size, 5 μm) using a gradient elution consisting of mobile phase A (0.1% formic acid in water) and mobile phase B (acetonitrile). The sample chamber in the autosampler was maintained at 4°C, whereas the column was set at 40°C. The gradient of the chromatographic condition was as follows: 0–5 min, linear gradient from 10 to 15% B; 5–15 min, linear gradient to 70% B; 15–18 min, linear gradient to 100% B; 18–23 min, 100% B; and 23–23.1 min, linear gradient back to 10% B. The entire analysis was completed in 30 min. PDA detection was performed from 200 to 400 nm. The 20 μL was applied as injection volume, and the flow rate was 0.2 mL/min. In the present study, M4 and M8 standards were not used.

The mass spectrometer was equipped with an electrospray ionization (ESI) source and operated in the positive mode. Mass spectrometric analyses were carried out by full-scan MS with a mass range of 10–500 Da and data-dependent MS/MS acquisition on the suspected metabolites ions. Liquid nitrogen was used as the nebulizing gas at a flow rate of 1.5 L/min. The capillary and skimmer voltages were set at 4.5 and 1.6 kV, respectively. The temperature of CDL and heat block remained at 200°C. The MS^2^ spectra were produced using collision-induced dissociation (CID) of the selected precursor ions using argon as collision gas with relative energy of 50%. The precursor ion isolation width at 1 Da and the ion accumulation time were set at 50 ms.

#### Data Acquisition and Processing

Prior to data acquisition, the external mass calibration was carried out using direct infusion of a reference standard from 50 to 1000 Da. The reference standard contained 0.1 g/L sodium hydrate and 0.25 mL/L trifluoroacetic acid. 5 μL/min was set as the flow rate of the infusion pump. After mass calibration with the reference standard, all calculated mass error was less than 5 ppm. The LC/MS solution version 3.41 software supplied with the instrument was used to data acquisition and processing. Any mass numbers corresponding to particular elemental composition were also calculated by the formula predictor, and would generate more than one formula proposed by the software. In the present study, an accuracy error threshold of ±20 ppm was set as a limit to the calculation of possible elemental compositions. The identification of MEQ and its metabolites were also based on the recent reports (Huang et al., 2015; Liu et al., 2017).

### Quantitative Real-Time PCR

The mRNA expression of genes related to BTB (e.g., *N*-cadherin, α-catenin, β-catenin, Connexin-43, CAR, F11R, Occludin and ZO-1) in mouse testes was measured. The levels of mRNA expression of the genes were determined by real-time quantitative reverse transcriptase-polymerase chain reaction (RT-PCR). Total RNA from the left testis from all males (*n* = 10) was extracted using the Trizol Reagent according to the manufacturer’s instructions. One microgram of RNA was reverse-transcribed to cDNA with the use of ReverTra Ace^TM^ First Strand cDNA Synthesis Kit (Promega, United States). Synthesized cDNA was used for RT-PCR (Bio-Rad, United States) with the SYBR^®^ Premix Ex Taq^TM^ RT-PCR kit (Takara, CodeDRR041 A, Japan).

The Primer Express Software was applied to design the mice specific primers according to the software guidelines (**Table 1**). Each 25 μL reaction mixture consisted of 12.5 μL SYBR^®^ Premix Ex Taq^TM^, 1.0 μL of each primer (10 μm), 2.0 μL of cDNA, and 8.5 μL RNase-Free H_2_O. For *N*-cadherin, α-catenin and β-catenin, the cycling conditions were as follows: step 1, 30 s at 95°C; step 2, 45 cycles at 95°C for 5 s, and 61°C for 30 s; and step 3, dissociation stage. For Connexin-43, F11R, Occludin and ZO-1, the cycling conditions were as follows: step 1, 30 s at 95°C; step 2, 45 cycles at 95°C for 5 s, and 60°C for 30 s; and step 3, dissociation stage. For CAR, the cycling conditions were as follows: step 1, 30 s at 95°C; step 2, 45 cycles at 95°C for 5 s, and 55°C for 30 s; and step 3, dissociation stage. In this study, the housekeeping gene β-actin was used as an internal calibrator reference gene for the expression profiling of genes.

**Table 1:** PCR primers used in the gene expression analysis.

Gene name	Description	Primer sequence (5′ - 3′)	Primer size (bp)
β-actin	mβ-actin-F	TTGCTGACAGGATGCAGAAG	141
	mβ-actin-R	ACATCTGCTGGAAGGTGGAC	
*N*-cadherin	mN-cadherin-F	ACGAAGGACAGCCCCTTCTCAA	87
	mN-cadherin-R	AATCTGCTGGCTCGCTGCTTTC	
α-catenin	mα-catenin-F	GGCGTGAAGCTTGTCCGAATGT	99
	mα-catenin -R	TTTACTCTGCGGCTTTGCTGCC	
β-catenin	mβ-catenin-F	GCCCAGCAAATCATGCGCCTTT	125
	mβ-catenin-R	CACAAACTGCTGCTGCGTTCCA	
Connexin-43	mConnexin-43-F	ATGCTGGTGGTGTCCTTGGTGT	86
	mConnexin-43-R	TTCACGCGATCCTTAACGCCCT	
F11R	mF11R-F	TTGGCTGGAACTACCGCATGGA	95
	mF11R-R	ACACACACGAGGCCACCAAAGA	
CAR	mCAR-F	ATTCCTGCTGACCGTTCTTG	145
	mCAR-R	GTCCGACAGTTTCTGCCATT	
Occludin	mOccludin-F	TAGTGGCTTTGGCTACGGAGGT	99
	mOccludin-R	AGGAAGCCTTTGGCTGCTCTTG	
ZO-1	mZO-1-F	ATGGAAAGCTGGGCTCTTGGCT	137
	mZO-1-R	ACCACCCGCTGTCTTTGGAAGT	


*The primers were manufactured by Nanjing Genscript Co., Ltd. (Nanjing, China). CAR, coxsackievirus and adenovirus receptor; F11R, Junctional adhesion molecule-A; ZO-1, zonula occludens-1.*

Following amplification, a melt curve analysis with the complementary computer software was applied to determine the authenticity of the amplified product described as its specific melting temperature (Tm). The threshold cycle of genes and the difference between their *C*t values (Δ*C*t) were counted. Relative quantitative analyses of gene expression were performed using 2^-ΔΔCt^ data analysis method in accordance with previous reports (Huang et al., 2009; Wang et al., 2011b, 2016c).

### Statistical Analysis

Statistical analysis was performed using SPSS 13.0 for Windows. All results are expressed as means ± SD. Group differences were assessed by one-way analysis of variance (ANOVA) followed by the least significance difference (LSD) test. *P* < 0.05 was considered statistically significant.

## Results

### Body Weight, Absolute Testes Weight and Relative Testis Weight

**FIGURE 2:**
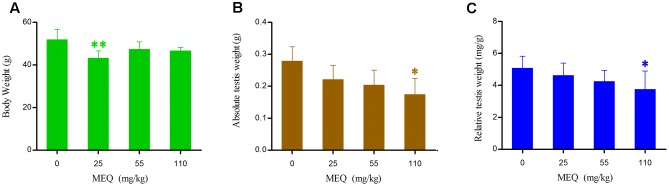
Body weight **(A)**, Absolute testis weight **(B)** and Relative testis weight **(C)** of male mice after the administration of MEQ for 18 months. ^∗^*p* < 0.05 in comparison with control, and ^∗∗^*p* < 0.01 in comparison with control. Values represent means ± SD (*n* = 10).

The final BW, absolute testis weight and relative testis weight of male mice after the administration of MEQ for 18 months are shown in **Figure 2**. The relative testis weight was expressed as milligrams (wet weight of testes mg)/(grams BW, g). Compared with the control group, the body and testis weights as well as relative testis weight were decreased in the MEQ treated groups. A significant reduction in absolute testis weight and relative testis weight were noted in the 110 mg/kg group (*p* < 0.05), and a significant decrease in BW was observed in the 25 mg/kg MEQ group (*p* < 0.01).

### Histological Evaluation

**FIGURE 3:**
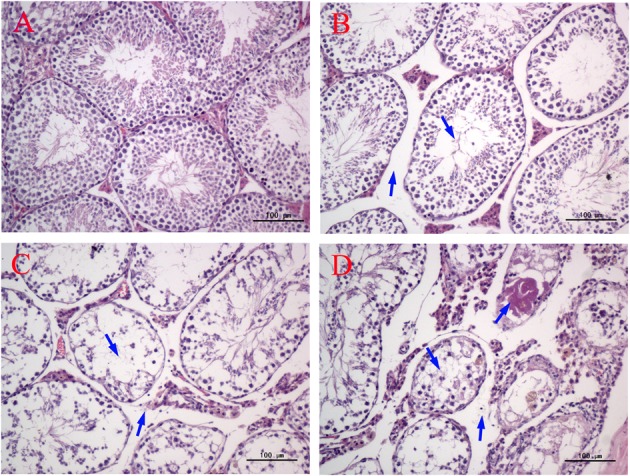
Effect of MEQ on the microphotographs of testes (200×). **(A)** Testes from the control group. Arrows show the testicular interstitial and seminiferous tubules are normal. **(B)** Testes from the 25 mg/kg MEQ group. Arrows show broadened interstitial testicular tissue, a few vacuoles in seminiferous tubules. **(C)** Testes from the 55 mg/kg MEQ group. Arrows show necrosis of the spermatogonia and spermatocytes in the lumen and the broadened interstitial testicular tissue. **(D)** Testes from the 110 mg/kg MEQ group. Arrows show seminiferous tubules with marked atrophy and red stained filamentous proteins (*n* = 5).

As shown in **Figure 3**, significant histopathological changes in testes were observed in half of the male mice of all the MEQ-treated groups. In the control testes, normal testicular interstitial and seminiferous tubules contained a larger number of developing sperm cells in the stratified epithelium and in the lumen of tubules (**Figure 3A**). The Sertoli cells and spermatogenic cells were arranged in an orderly manner in the seminiferous tubules (**Figure 3A**). Compared with the control group, with broadened interstitial testicular tissue, a few vacuoles in seminiferous tubules, and the irregular arrangement and decreased number as well as the reduced layers of sperm spermatogenic cells in lumen were found in mice treated with 25 mg/kg (**Figure 3B**). The most obvious damage to testicular structure was found in the 55 and 110 mg/kg groups. The spermatogonia and spermatocytes in the lumen showed necrosis, while the residual spermatocytes entered the central lumen. The sperm in the lumen disappeared in most seminiferous tubules (**Figure 3C**). Serious pathological changes in seminiferous tubule cells included the disorganization of germinal epithelium, atrophy of the seminiferous tubule, and completely absent spermatogenic cells with the left red stained filamentous protein (**Figure 3D**). The significant reduction in the number of sperm and the enlarged of testicular interstitial indicated the destruction of BTB in testes after chronic exposure to MEQ.

### Ultrastructural Alterations

**FIGURE 4:**
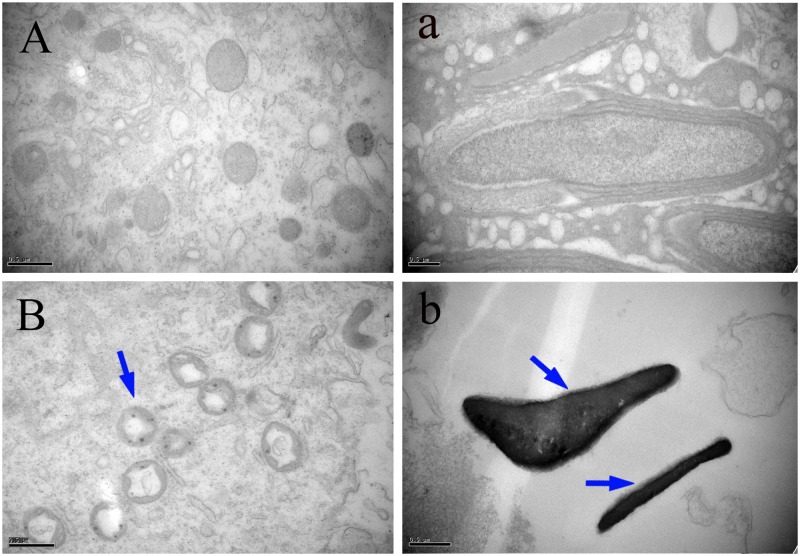
Effect of MEQ on the ultrastructure of mitochondria and sperm under TEM (Scale bar = 0.5 μm). **(A)** Mitochondria from the control group. **(B)** Mitochondria from the 110 mg/kg MEQ group. Arrow shows swelling, vacuoles and loss of crests. **(a)** Sperm from the control group. **(b)** Sperm from the 110 mg/kg MEQ group. Arrows show abnormal morphology, rupture and dissolution of the membrane.

**FIGURE 5:**
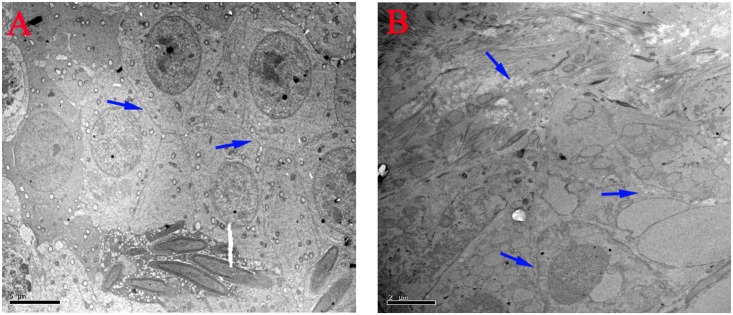
Effect of MEQ on the ultrastructure of BTB under TEM (Scale bar = 5 μm, Scale bar = 2 μm). **(A)** BTB from the control group. **(B)** BTB from the 110 mg/kg. Arrows show the destroyed integrity of the BTB.

The ultrastructural changes of the mitochondria, sperm morphology and BTB in the testes of male mice after the administration of 25, 55, and 110 mg/kg MEQ for 18 months were studied by TEM. The mitochondria exhibited swelling, vacuoles, loss of crests, and damaged membrane structure in the 110 mg/kg group (**Figure 4B**). Sperm in mice testes from the control group exhibited integral membranes and normal sizes (**Figure 4a**). In the 110 mg/kg group, the sperm showed an obvious abnormal morphological structure with dissolving membranes. Additionally, as shown in **Figure 5**, there were no visible BTB-associated junctions in the 110 mg/kg group (**Figure 5B**). This finding indicated that the mitochondrial damage, sperm abnormity and destroyed integrity of BTB were involved in the reproductive toxicity mediated by MEQ.

### Oxidative Stress Indices

**FIGURE 6:**
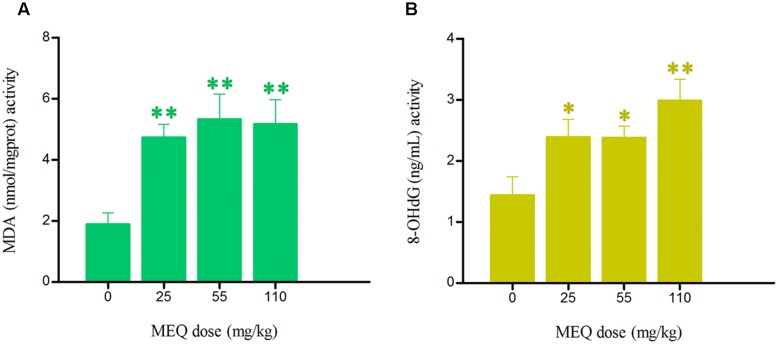
Effects of MEQ on **(A)** Lipid peroxidation (MDA) and **(B)** Peroxidation of DNA (8-OHdG) in the serum of male mice after the administration of MEQ for 18 months. ^∗^*p* < 0.05 in comparison with control, and ^∗∗^*p* < 0.01 in comparison with control. Values represent means ± SD (*n* = 10).

The effect of MEQ on the levels of 8-OHdG and MDA in serum of male mice was shown in **Figure 6**. Compared with the control group, significant increased levels of MDA in the serum were markedly elevated at all MEQ treated groups (*p* < 0.01). The remarkable induced levels of 8-OHdG were noted at 25 and 55 mg/kg groups (*p* < 0.05), and 110 mg/kg group (*p* < 0.01). These results show that the oxidative stress was triggered in male mice after chronic administration of MEQ.

### MEQ and Its Metabolites in Serum and Testes

To verify the hypothesis that MEQ may pass through the BTB into the testes to cause reproductive toxicity, we identified MEQ and its metabolites in serum and testis by LC/MS-IT-TOF analysis according to the retention times and fragment ions. As showed in Supplementary Figure S1, the accurate extracted mass chromatograms (EICs) of MEQ standard was similar to those in control samples (serum and testis) that were added to MEQ standard, indicating that the pretreatment methods of serum and testis can’t affected the structure of MEQ, which was consistent with the previous studies (Liu Z.Y. et al., 2010; Ihsan et al., 2011).

**FIGURE 7:**
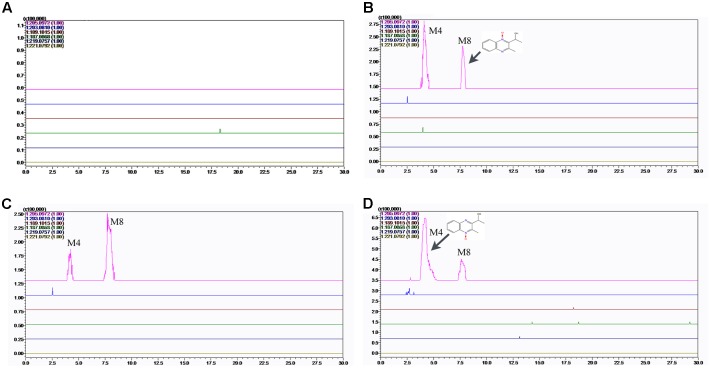
Accurate EIC of MEQ and metabolites of MEQ in the serum of male mice. **(A)** Serum of control. **(B)** Serum of 25 mg/kg MEQ. **(C)** Serum of 55 mg/kg MEQ. **(D)** Serum of 110 mg/kg MEQ.

**FIGURE 8:**
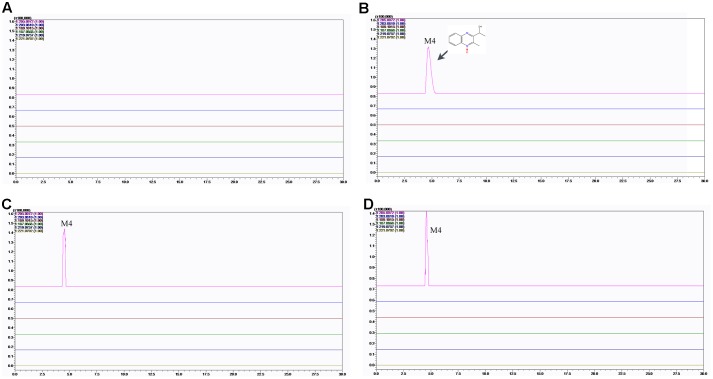
Accurate EIC of MEQ and metabolites of MEQ in the testes of male mice. **(A)** Testis of the control. **(B)** Testis from the 25 mg/kg MEQ group. **(C)** Testis from the 55 mg/kg MEQ group. **(D)** Testis from the 110 mg/kg MEQ group.

The predicated elemental composition, measured masses, exact masses, and mass errors were indicated in **Table 2**. The mass errors between measured and predicated masses of M4 and M8 were 3.4 and 4.9 ppm, respectively, which agree to within less than 6 ppm, providing support for the proposed elemental composition of the metabolites. The retention times and fragment ions information used in the identification of M4 and M8 were summarized in **Table 3**. Metabolites of M4 and M8 showed [M+H]^+^ ions at m/z 205, and had retention times of 4.5 and 7.7 min, respectively. As shown in **Figures 7**, **8**, MEQ showed protonated molecular ion at m/z 219. This result demonstrated a loss of 14 Da (m/z 219-205) of M4 and M8 from MEQ, suggesting that M4 and M8 were *N*→O group reduction (loss of 16 Da) and side chain hydrogenation (acquisition 2 Da) of MEQ. The difference between M4 and M8 lay in the position of *N*→O group reduction in MEQ.

**Table 2:** The predicated elemental composition, measured masses, exact masses, and mass errors between measured and predicated masses of M4 and M8.

Metabolite	Elemental composition ([M+H]^+^)	Measured masse (Da)	Exact masse (Da)	Error (mDa)	Error (ppm)
M4	C_11_H_13_N_2_O_2_^+^	205.0979	205.0972	0.7	3.4
M8	C_11_H_13_N_2_O_2_^+^	205.0982	205.0972	1.0	4.9


**Table 3:** The retention times (RT) and fragment ions of M4 and M8.

Metabolite	RT (min)	[M+H]+ (m/z)	Major fragment ions	Identification
M4	4.5	205	188, 146	3-methyl-2-(1-hydroxyethyl) quinoxaline-*N*4-monoxide
M8	7.7	205	188, 170	3-methyl-2-(1-hydroxyethyl) quinoxaline-*N*1-monoxide


**FIGURE 9:**
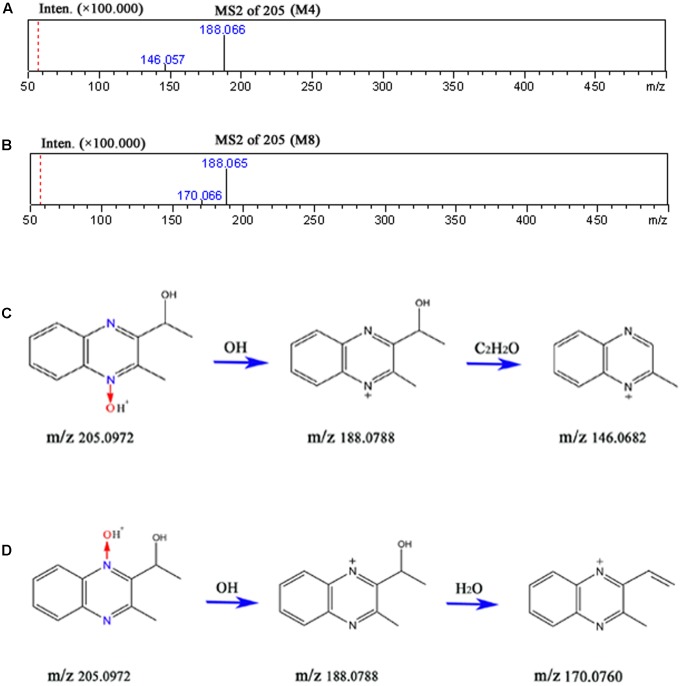
The accurate MS^2^ spectral for M4 **(A)** and M8 **(B)**, and proposed fragmentation pathways of M4 **(C)** and M8 **(D)**.

The accurate MS^2^ spectral for M4 and M8, and proposed fragmentation pathways of M4 and M8 were presented in **Figure 9**. M4 lost HO^•^ radical (observed 17.0032 Da, predicated 17.0028) to form m/z 188 following loss of C_2_H_2_O (observed 42.0022 Da, predicated 42.0013) to form m/z 146 (**Figure 9C**). M8 lost HO^•^ radical (observed 17.0032 Da, predicated 17.0028) to form m/z 188 (**Figure 9D**). The fragment ions at m/z 170 was formed by the loss of H_2_O (observed 18.0022 Da, predicated 18.0013) from m/z 188 (**Figure 9D**). The fragments and retention times of M4 and M8 detected in our study were consistent with those identified in the pigs and chickens after administration of [^3^H]-MEQ by oral gavage (Huang et al., 2015). Therefore, M4 and M8 were confirmed as M4, and M8, respectively.

The results showed that no MEQ was found in the serum and testis. Two metabolites (M4 and M8) were detected in the serum in MEQ exposure groups (**Figure 7**), while only one metabolite (M4) was found in the testis after the administration of MEQ for 18 months (**Figure 8**). These results indicated that M4 may be the toxic metabolite in the testes of mice that could pass through BTB to induce sperm aberration and reproductive toxicity.

### The Expressions of BTB Related Genes

**FIGURE 10:**
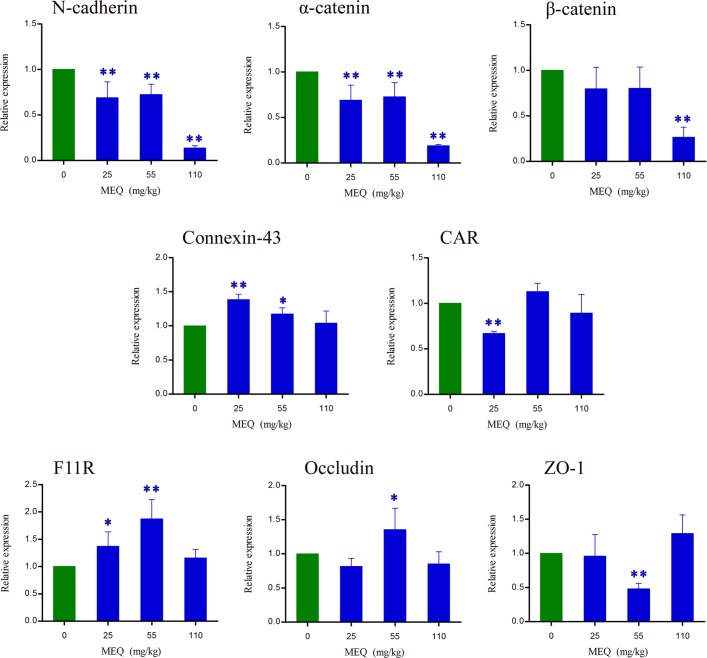
Alterations of *N*-cadherin, α-catenin, β-catenin, Connexin-43, CAR, F11R, Occludin and ZO-1 expression in mouse testes after the administration of MEQ for 18 months. ^∗^*p* < 0.05 in comparison with control, and ^∗∗^*p* < 0.01 in comparison with control. Values represent means ± SD (*n* = 10).

To confirm whether BTB is involved in MEQ-induced male reproductive toxicity in male mice, the mRNA expression of some BTB related genes (e.g., *N*-cadherin, α-catenin, β-catenin, Connexin-43, CAR, F11R, Occludin and ZO-1) were evaluated using RT-PCR (**Figure 10**). Exposure to MEQ significantly reduced the expression of *N*-cadherin and α-catenin in all of the treated groups (*p* < 0.01). Decreased mRNA expression of CAR, ZO-1 and β-catenin was observed in 25, 55, and 110 group, accordingly (*p* < 0.01). There was a marked increased level of Connexin-43 and F11R expression in both 25 and 55 mg/kg MEQ groups (*p* < 0.05 or *p* < 0.01). A significant increase in the expression level of Occludin was noted in the 55 mg/kg group (*p* < 0.05). These results support the findings of ultrastructural alterations at the highest dose level and indicate potential effects also on the structure and integrity of BTB at lower doses.

## Discussion

Previous studies have illustrated that oxidative stress and metabolites are associated with the *in vitro* toxicity in H295R cells (Wang et al., 2016c), and *in vivo* toxicity in rats (Huang et al., 2009; Ihsan et al., 2011; Wang et al., 2011b) and mice (Liu et al., 2017). However, relatively little is known about the role of these two factors in the reproductive toxicity induced by MEQ *in vivo*. Although it has been documented that MEQ exposure induced male reproductive toxicity (Ihsan et al., 2011), it still remains unclear whether the testis is the primary cellular target for MEQ mediated-male reproductive toxicity. In the present study, the results demonstrated that the metabolites of MEQ, oxidative stress, mitochondrion dysfunction and spermatogenesis deficiency are involved in the reproductive toxicity in mice after the chronic administration of MEQ for up to 18 months. Additionally, it was suggested that M4, with higher reproductive toxicity than M8, might pass through the BTB to cause direct damage to the testes. Moreover, the adverse effect of toxic metabolites of MEQ on the testis was first noted, and the disrupted BTB further resulted in the invasion of M4 into the testes to invoke the subsequent abnormal spermatogenesis.

It has been documented that MEQ exposure induced reproductive toxicity in rats. Ihsan et al. (2011) reported that a significant reduction in the final body and testicular weight and a significant increase in the relative testicular weight were observed on Wistar rats. MEQ was found to change sperm morphology, decrease fertility, and alter the growth and development of the next generation (Ihsan, 2011). In the present study, a reduction in BW, absolute testis weight, and the relative testis weight were observed when male mice were administered MEQ at doses of 25, 55, and 110 mg/kg for an extended period of time (**Figure 2**). Herein, a reduction in BW suggested that even low dose of MEQ showed its toxicity after long exposure to mice. The histopathological evaluation of MEQ-treated mice showed marked testis damage, including broadened interstitial testicular tissue, a few vacuoles in the seminiferous tubules, and the necrosis of both spermatogonia and spermatocytes in the lumen (**Figure 3**). Mitochondria are best known as multitasking organelles that participate in various cellular functions, such as ATP production, calcium homoeostasis, the generation of ROS, the intrinsic apoptotic pathway and steroid hormone biosynthesis (Amaral et al., 2013). Scientific reports have revealed that the changes in mitochondrial integrity/functionality will result in physiological dysfunction, including infertility and the loss of sperm function (particularly with decreased motility) (Pena et al., 2009, 2015; Amaral et al., 2013; Plaza Davila et al., 2015). In the MEQ-treated groups, the mitochondria exhibited swelling, vacuoles, loss of crests, and absolute dissolved mesenchyme in TEM analysis (**Figure 4**). Therefore, the obvious ultrastructural changes in the mitochondria in the present study might be responsible for the deficiency in spermatogenesis caused by MEQ. Additionally, the obvious aberration and dissolution of sperm as well as the destroyed integrity of BTB were noted when male mice were exposed to MEQ for 18 months (**Figures 4**, **5**). Here, we not only proved the earlier findings that testis damage occurs after exposure to MEQ (Ihsan et al., 2011), but also firstly found that MEQ induced adverse effect on the mitochondrial and BTB, and caused toxicity to spermatogenesis, sperm development and sperm maturation.

It was illustrated that oxidative stress resulted in the serious damage to cellular macromolecules, such as DNA, lipids and proteins by causing disorder of the antioxidant defense system (Wang et al., 2015a). Herein, our results showed that the levels of MDA and 8-OHdG were significantly increased in the MEQ-treated groups (**Figure 5**), suggesting an imbalance of redox in male mice. The generation of ROS and unstable oxygen-sensitive radical intermediates of QdNOs were considered critical in DNA damage (Ganley et al., 2001; Poole et al., 2002; Junnotula et al., 2009; El-Khatib et al., 2010; Cheng et al., 2015). A lot of evidence suggested that the *in vivo* toxicity mediated by QdNOs was associated with oxidative stress. As the earlier findings suggested, the production of oxidative stress during the *N*→O reduction of QdNOs, was found to be a main factor in the QdNOs-mediated toxicity in testicular (Ihsan et al., 2011), spleen (Wang et al., 2011b), adrenal (Huang et al., 2009; Wang et al., 2016a,c), and liver (Wang et al., 2011b; Zhao et al., 2011; Liu et al., 2017), as well as genotoxicity (Wang et al., 2016d). Therefore, our data demonstrated that the oxidative stress triggered by MEQ may exhibit the toxicity to mitochondria, which is involved in the dysfunction of the reproductive system mediated by MEQ.

**FIGURE 11:**
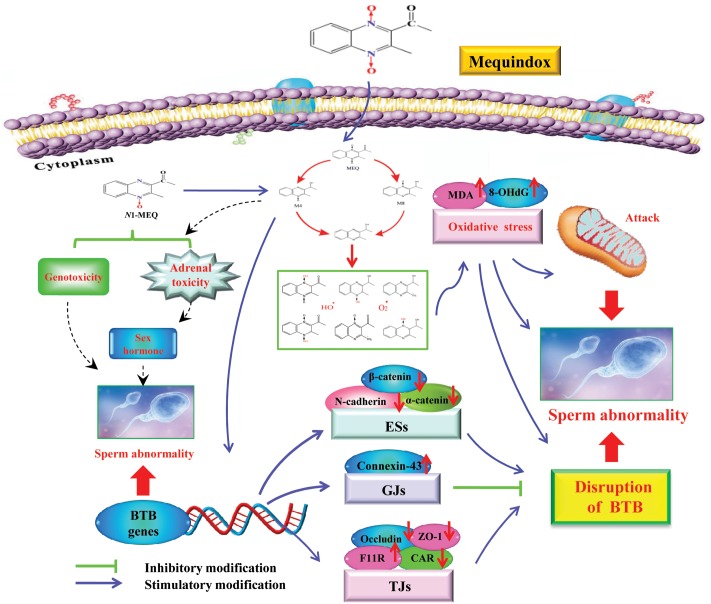
The proposed mechanisms of reproductive toxicity in mouse caused by MEQ. The oxidative damage occurs via reduction of the *N*→O group of MEQ, which causes mitochondrial dysfunction and sperm abnormality. M4 passed through the BTB with a disruptive effect on BTB via alteration of the related genes (e.g., TGs, GJs and basal ESs). The disrupted BTB further resulted in the invasion of M4 into the testes to exert the toxic effect on the mitochondria and spermatogenic cells. The adrenal toxicity and genotoxicity caused by *N*1-MEQ, may contribute to sperm abnormalities.

A lot of evidence suggested that the major metabolic pathway for QdNOs involves *N*→O group reduction (Cheng et al., 2015, 2016; Liu Q. et al., 2016), and that this type of metabolism is closely related to their toxicity (Hao et al., 2006; Chen et al., 2008, 2009). In this study, two metabolites of M4 and M8 were detected in the serum of male mice by LC/MS-IT-TOF analysis (**Figure 7**), which directly confirms the potential connection of *N*→O group reduction metabolism of MEQ with its organ toxicity. As many studies suggested, the toxicity of QdNOs was associated with their metabolites when these substances were extensively metabolized *in vivo* (Huang et al., 2009, 2010; Liu et al., 2017), and therefore, it comes important to understand the toxic metabolites, species differences and amount of toxic metabolites. The quantify of the toxic metabolites should be considered when determined the no-observed-adverse-effect-level (NOAEL) for reproductive toxicity of MEQ. However, the amount of the metabolites can’t be made due to the M4 and M8 standards were not used in this study. Further study should be conducted to make the toxic metabolites of MEQ *in vivo* quantitative. M4 and M8 were recognized as toxic metabolites in mice liver after the administration of MEQ for 11 months (Liu et al., 2017). In general, the drug is metabolized in the liver and then distributed into the blood. Here, the occurrence of M4 and M8 in the serum of male mice may be derived from the specific metabolic pathway of MEQ in the male liver under pathological conditions. Our recent study suggested that the specific metabolic pathway was considered an important consequence for the alteration of drug-metabolizing enzymes in response to the imbalance oxidative stress (Liu et al., 2017). Therefore, during *N*→O group reduction of MEQ, the highly reactive radicals including MEQ radical intermediates, as well as O_2_^•-^ and HO^•^, were generated and subsequently caused oxidative stress in mouse (**Figure 11**). These findings further confirmed our previous report of the proposed metabolic pathway of MEQ in mice, suggesting the critical role of the metabolism in organ toxicity induced by MEQ.

Blood-testis barrier, one of the tightest blood-tissue barriers, is crucial to male fertility as it physically divides the seminiferous epithelium into basal and apical compartments (Saunders, 2003; Liu W.Y. et al., 2012; Lu et al., 2016). The BTB prevents the diffusion of various endogenous and exogenous toxic substances from entering apical compartments, and was therefore considered as one of the most important pathways related to chemically induced reproductive toxicity (Siu et al., 2009; Lu et al., 2016; Zhang et al., 2016). The integrity of the BTB is based on various junctions, forming TJs, GJs and basal ESs, to provide structural and nutritional support to germ cells (Cheng et al., 2011; Zhang et al., 2016). The immunological and “fence” function of BTB is to facilitate spermiogenesis and spermiation during the epithelial cycle; dysfunction of the BTB will result in the disruption of spermatogenesis and infertility (Cheng and Mruk, 2012; Qiu et al., 2013). Regarding the reproductive toxicity induced by MEQ, only one study has been conducted, which identified M11, an *N*→O reduction metabolite of MEQ, in the testis of wistar rats accompanying oxidative stress (Ihsan et al., 2011), suggesting that M11 may pass through the BTB. M11 is factually the same metabolite than M8 identified in the present study. However, relatively little is known about whether the testis is one of the main target organs of MEQ. Furthermore, it has been well recognized that there are generally species-specific differences in the metabolism of drugs. Thus, potential toxic metabolites of MEQ in the testis of male mice, and the role of BTB-associated junctions in reproductive toxicity after exposure to MEQ still need to be illustrated.

To investigate whether the testis is the primary cellular target for MEQ to induce reproductive toxicity, and whether MEQ could pass through the BTB, we identified the MEQ and its metabolites in the testes of male mice by LC/MS-IT-TOF analysis. In a study of the relationship between the metabolites of MEQ and liver damage, the M4 and M8 were determined in the liver of mice accompanying the oxidative stress (Liu et al., 2017). Therefore, the M4 and M8 could be regarded as a biomarker of liver damage induced by MEQ. Herein, the M4 detected in the testes indicated a potential biomarker of reproductive toxicity caused by MEQ in male mice (**Figure 8**). Therefore, apart from oxidative stress, the metabolite of M4 was another main factor in reproductive toxicity and the changes in testicular structure. M4 was a hydroxylated product of the side chain of *N*1-MEQ, a partially reduced derivative of MEQ (Liu and Sun, 2013; Huang et al., 2015; Liu Q. et al., 2016). Previous studies revealed that *N*1-MEQ exhibited adrenal toxicity in H295R cells (Wang et al., 2016c) and genotoxicity in mice (Liu Q. et al., 2016). The connections of sperm abnormality with genotoxicity (Jagtap et al., 2014; Devaux et al., 2015; Dong et al., 2015; Das et al., 2016; Liu W. et al., 2016) and sex hormones (Liu W. et al., 2016; Cremonese et al., 2017; Ring et al., 2017) were well recognized. Commonly, alterations of sex hormones and steroidogenic pathways are associated with the function of the adrenal gland (Sohn et al., 2016). Therefore, apart from oxidative stress, the genotoxicity and adrenal toxicity may also participate in the sperm morphology, as evidenced by our observations in TEM analysis (**Figure 11**).

**FIGURE 12:**
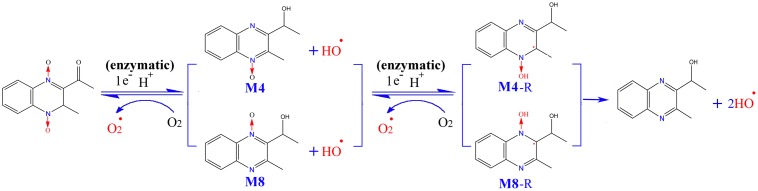
The proposed metabolic pathways of MEQ in male mouse to better understand the higher reproductive toxicity of M4 compared to M8. The production of M4-R and M8-R intermediate radicals, as well as O_2_^•-^ and OH^•^ occurs via reduction of the *N*→O group of MEQ. Close to an electron on the quinoxaline ring, a methyl and a hydroxyl were found in M4-R, while two hydroxyl groups were noted in M8-R.

Interestingly, there are two metabolites of MEQ (M4 and M8) detected in the serum, while only M4 could pass through the BTB and appear in the testis, suggesting a higher degree of reproductive toxicity of M4 than M8. Besides the traditional O_2_^•-^ and HO^•^, the crucial oxygen-sensitive radical intermediates during the metabolism of QdNOs were found to be closely related to mutagenicity (Ganley et al., 2001), antibacterial activity (Cheng et al., 2015) and hypoxic cytotoxin (Junnotula et al., 2009; El-Khatib et al., 2010) caused by QdNOs. Additionally, the varying degree of toxicity of QdNOs contributed to the lasting-time of ROS (Wang et al., 2015b) and the stability of the radical intermediates (El-Khatib et al., 2010). Therefore, the stability of the radical intermediates plays a critical role in the toxicity induced by MEQ *in vivo* (Liu et al., 2017). M4 and M8 were generated from MEQ via intracellular single deoxidation and the hydroxylation of side chains, accompanying the production of ROS including O_2_^•-^ and HO^•^ (**Figure 12**). Then, M4 and M8 undergo one-electron enzymatic reduction to yield highly reactive intermediates radicals of M4-R and M8-R, respectively. As is already known, methyl and hydroxyl are electron donors and electron-withdrawing groups, respectively. Close to an electron on a quinoxaline ring, a methyl and a hydroxyl were noted in M4-R, while two hydroxyl groups were seen in M8-R, suggesting a uniform distribution of the electron cloud in M4-R. Therefore, M4-R, which resulted from the addition of an electron to C3 of MEQ, is more stable, by resonance, and causes longer lasting and increased damage to the testis than M8-R, resulting from the addition of an electron to the C2 of MEQ. This may be an important reason for the higher reproductive toxicity of M4 than M8. Furthermore, this finding throws new light onto the drug intermediate radicals and organ toxicity of QdNOs.

To further reveal the possible mechanism responsible for the disruption of BTB caused by M4, the mRNA expression levels of BTB-associated junctions in the testis were evaluated. The results demonstrated that after exposure to MEQ for 18 months, the following mRNA expression of BTB-associated junctions was down-regulated: (1) basal ESs mRNA: *N*-cadherin, α-catenin and β-catenin; and (2) TJ integral membrane mRNA: CAR (25 and 110 mg/kg groups), Occludin (25 and 110 mg/kg groups) and ZO-1 (25 and 55 mg/kg groups) (**Figure 10**), while the mRNA expression of F11R and connexin-43, which belongs to TJs and GJs, respectively, were increased in all MEQ exposure groups (**Figure 10**). Although there is lack of (clear) dose response, the results are considered relevant as many factors may have contributed on the expression of mRNA. These findings indicated that the high level of F11R and connexin-43 expression may be a cellular protective response to MEQ reproductive toxicity (**Figure 11**). The integrity of the BTB is based on various junctions including basal ESs (*N*-cadherin, α-catenin and β-catenin), GJs (Connexin-43), and TJs (CAR, F11R, Occludin and ZO-1), to provide structural and nutritional support to germ cells (Cheng et al., 2011; Zhang et al., 2016). The immunological and “fence” function of BTB is to facilitate spermiogenesis and spermiation during the epithelial cycle (Cheng and Mruk, 2012; Qiu et al., 2013). Therefore, the disruptive effect of MEQ on BTB was initially mediated by suppression of the basal ESs and TJs-mRNA expression (**Figure 11**). The current result also suggested that the basal ESs and TJs might be the targets for M4 and the destroyed BTB further resulted in the invasion of M4 and other harmful substances into the testes to exert toxic effects on mitochondrion and spermatogenic cells.

## Conclusion

The present study confirmed that M4 and M8 were potentially major toxic metabolites in oxidative damage *in vivo*, and demonstrated that the oxidative stress and metabolite M4 were involved in the reproductive toxicity in male mice after the administration of MEQ for 18 months. M4 passed through the BTB to interfere in spermatogenesis with the TJ- and basal ES-associated junctions as macromolecular targets. The higher reproductive toxicity of M4 than M8 might derive from the increased stability of M4-R than the radical that resulted from the M8. It is fascinating to pay more attention to the relationship between the oxidative stress, intermediate radicals and organ toxicity mediated by QdNOs. The genotoxicity and adrenal toxicity may contribute to the sperm abnormity caused by MEQ. Moreover, the current study also revealed that the mitochondrial dysfunction and spermatogenesis deficiency were induced by MEQ, and the further study needs to be conducted to investigate the underlying molecular mechanism.

## Author Contributions

ZY conceived the idea. XW analyzed and discussed data. QyL analyzed and discussed data and wrote the paper. ZL performed and revised the experiments. AH and QrL performed the experiments. IA and SA revised the paper. All the authors discussed the results and contributed to the final manuscript.

## Conflict of Interest Statement

The authors declare that the research was conducted in the absence of any commercial or financial relationships that could be construed as a potential conflict of interest.

## Abbreviations

8-OHdG: 8-hydroxydeoxyguanosine
BTB: blood-testis barrier
CBX: carbadox
ESs: ectoplasmic specializations
GJs: gap junctions
LC/MS-IT-TOF: high-performance liquid chromatography-mass spectrometry-ion trap-time-of-flight
M4: 3-methyl-2-(1-hydroxyethyl) quinoxaline-*N*4-monoxide
M4-R: M4 radical
M8: 3-methyl-2-(1-hydroxyethyl) quinoxaline-*N*1-monoxide
M8-R: M8 radical
MDA: malondialdehyde
MEQ: mequindox
*N*1-MEQ: *N*1-desoxymequindox
OLA: olaquindox
QCT: quinocetone
QdNOs: quinoxaline-di-*N*-oxides
ROS: reactive oxygen species
TEM: Transmission electron microscope
TJs: tight junctions

## References

[B1] Amaral A. Lourenco B. Marques M. Ramalho-Santos J. (2013). Mitochondria functionality and sperm quality. *Reproduction* 146 163–174. 10.1530/REP-13-0178 23901129

[B2] Azqueta A. Arbillaga L. Pachon G. Cascante M. Creppy E. E. Lopez de Cerain A. (2007). A quinoxaline 1,4-di-N-oxide derivative induces DNA oxidative damage not attenuated by vitamin C and E treatment. *Chem. Biol. Interact.* 168 95–105. 10.1016/j.cbi.2007.02.013 17420013

[B3] Chen Q. Chen Y. Qi Y. Hao L. Tang S. Xiao X. (2008). Characterization of carbadox-induced mutagenesis using a shuttle vector pSP189 in mammalian cells. *Mutat. Res.* 638 11–16. 10.1016/j.mrfmmm.2007.08.006 17897684

[B4] Chen Q. Tang S. S. Jin X. Zou J. J. Chen K. P. Zhang T. (2009). Investigation of the genotoxicity of quinocetone, carbadox and olaquindox in vitro using Vero cells. *Food Chem. Toxicol.* 47 328–334. 10.1016/j.fct.2008.11.020 19061932

[B5] Cheng C. Y. Mruk D. D. (2012). The blood-testis barrier and its implications for male contraception. *Pharmacol. Rev.* 64 16–64. 10.1124/pr.110.002790 22039149 PMC3250082

[B6] Cheng C. Y. Wong E. W. Lie P. P. Li M. W. Mruk D. D. Yan H. H. (2011). Regulation of blood-testis barrier dynamics by desmosome, gap junction, hemidesmosome and polarity proteins: an unexpected turn of events. *Spermatogenesis* 1 105–115. 10.4161/spmg.1.2.15745 22319658 PMC3271652

[B7] Cheng G. Li B. Wang C. Zhang H. Liang G. Weng Z. (2015). Systematic and molecular basis of the antibacterial action of Quinoxaline 1,4-Di-N-Oxides against *Escherichia coli*. *PLOS ONE* 10:e0136450 10.1371/journal.pone.0136450 PMC4546592 26296207

[B8] Cheng G. Sa W. Cao C. Guo L. Hao H. Liu Z. (2016). Quinoxaline 1,4-di-N-Oxides: biological activities and mechanisms of actions. *Front. Pharmacol.* 7:64 10.3389/fphar.2016.00064 PMC4800186 27047380

[B9] Chowdhury G. Kotandeniya D. Daniels J. S. Barnes C. L. Gates K. S. (2004). Enzyme-activated, hypoxia-selective DNA damage by 3-amino-2-quinoxalinecarbonitrile 1,4-di-N-oxide. *Chem. Res. Toxicol.* 17 1399–1405. 10.1021/tx049836w 15540937

[B10] Cremonese C. Piccoli C. Pasqualotto F. Clapauch R. Koifman R. J. Koifman S. (2017). Occupational exposure to pesticides, reproductive hormone levels and sperm quality in young Brazilian men. *Reprod. Toxicol.* 67 174–185. 10.1016/j.reprotox.2017.01.001 28077271

[B11] Dai C. Tang S. Li D. Zhao K. Xiao X. (2015). Curcumin attenuates quinocetone-induced oxidative stress and genotoxicity in human hepatocyte L02 cells. *Toxicol. Mech. Methods* 25 340–346. 10.3109/15376516.2015.1045659 25996037

[B12] Das S. Upadhaya P. Giri S. (2016). Arsenic and smokeless tobacco induce genotoxicity, sperm abnormality as well as oxidative stress in mice in vivo. *Genes Environ.* 38:4 10.1186/s41021-016-0031-2 PMC4917979 27350824

[B13] Devaux A. Bony S. Plenet S. Sagnes P. Segura S. Suaire R. (2015). Field evidence of reproduction impairment through sperm DNA damage in the fish nase (*Chondrostoma nasus*) in anthropized hydrosystems. *Aquat. Toxicol.* 169 113–122. 10.1016/j.aquatox.2015.10.013 26523677

[B14] Dong J. Wang Z. Zou P. Zhang G. Dong X. Ling X. (2015). Induction of DNA damage and G2 cell cycle arrest by diepoxybutane through the activation of the Chk1-dependent pathway in mouse germ cells. *Chem. Res. Toxicol.* 28 518–531. 10.1021/tx500489r 25633853

[B15] El-Khatib M. Geara F. Haddadin M. J. Gali-Muhtasib H. (2010). Cell death by the quinoxaline dioxide DCQ in human colon cancer cells is enhanced under hypoxia and is independent of p53 and p21. *Radiat. Oncol.* 5:107 10.1186/1748-717X-5-107 PMC2993715 21078189

[B16] Ganley B. Chowdhury G. Bhansali J. Daniels J. S. Gates K. S. (2001). Redox-activated, hypoxia-selective DNA cleavage by quinoxaline 1,4-di-N-oxide. *Bioorg. Med. Chem.* 9 2395–2401. 10.1016/S0968-0896(01)00163-8 11553481

[B17] GB15193.17 (2003). *Chronic Toxicity and Carcinogencity Study.* Haidian District: National Institute of Standards of the People’s Republic of China, 109–113.

[B18] Hao L. Chen Q. Xiao X. (2006). Molecular mechanism of mutagenesis induced by olaquindox using a shuttle vector pSP189/mammalian cell system. *Mutat. Res.* 599 21–25. 10.1016/j.mrfmmm.2005.12.017 16513143

[B19] Huang L. Yin F. Pan Y. Chen D. Li J. Wan D. (2015). Metabolism, distribution, and elimination of mequindox in pigs, chickens, and rats. *J. Agric. Food Chem.* 63 9839–9849. 10.1021/acs.jafc.5b02780 26376954

[B20] Huang X. J. Ihsan A. Wang X. Dai M. H. Wang Y. L. Su S. J. (2009). Long-term dose-dependent response of Mequindox on aldosterone, corticosterone and five steroidogenic enzyme mRNAs in the adrenal of male rats. *Toxicol. Lett.* 191 167–173. 10.1016/j.toxlet.2009.08.021 19733641

[B21] Huang X. J. Zhang H. H. Wang X. Huang L. L. Zhang L. Y. Yan C. X. (2010). ROS mediated cytotoxicity of porcine adrenocortical cells induced by QdNOs derivatives in vitro. *Chem. Biol. Interact.* 185 227–234. 10.1016/j.cbi.2010.02.030 20188712

[B22] Ihsan A. (2011). *Preclinical Toxicology of Mequindox.* Ph.D. thesis, Huazhong Agricultural University, Wuhan.

[B23] Ihsan A. Wang X. Huang X. J. Liu Y. Liu Q. Zhou W. (2010). Acute and subchronic toxicological evaluation of Mequindox in Wistar rats. *Regul. Toxicol. Pharmacol.* 57 307–314. 10.1016/j.yrtph.2010.03.011 20371258

[B24] Ihsan A. Wang X. Liu Z. Wang Y. Huang X. Liu Y. (2011). Long-term mequindox treatment induced endocrine and reproductive toxicity via oxidative stress in male Wistar rats. *Toxicol. Appl. Pharmacol.* 252 281–288. 10.1016/j.taap.2011.02.020 21377486

[B25] Ihsan A. Wang X. Tu H.-G. Zhang W. Dai M.-H. Peng D.-P. (2013). Genotoxicity evaluation of Mequindox in different short-term tests. *Food Chem. Toxicol.* 51 330–336. 10.1016/j.fct.2012.10.003 23063596

[B26] Jagtap C. Y. Chaudhari S. Y. Thakkar J. H. Galib R. Prajapati P. K. (2014). Assessment of genotoxic potential of hridayarnava rasa (a herbo-mineralo-metallic ayurvedic formulation) using chromosomal aberration and sperm abnormality assays. *Toxicol. Int.* 21 242–247. 10.4103/0971-6580.155331 25948961 PMC4413405

[B27] Junnotula V. Sarkar U. Sinha S. Gates K. S. (2009). Initiation of DNA strand cleavage by 124-benzotriazine 14-dioxide antitumor agents: mechanistic insight from studies of 3-methyl-124-benzotriazine 14-dioxide. *J. Am. Chem. Soc.* 131 1015–1024. 10.1021/ja8049645 19117394 PMC2819123

[B28] Li G. Yang F. He L. Ding H. Sun N. Liu Y. (2012). Pharmacokinetics of mequindox and its metabolites in rats after intravenous and oral administration. *Res. Vet. Sci.* 93 1380–1386. 10.1016/j.rvsc.2012.02.015 22459092

[B29] Li J. Huang L. L. Wang X. Pan Y. H. Liu Z. Y. Chen D. M. (2014). Metabolic disposition and excretion of quinocetone in rats, pigs, broilers, and carp. *Food Chem. Toxicol.* 69 109–119. 10.1016/j.fct.2014.04.004 24727229

[B30] Liu J. Ouyang M. Jiang J. Mu P. Wu J. Yang Q. (2012). Mequindox induced cellular DNA damage via generation of reactive oxygen species. *Mutat. Res.* 741 70–75. 10.1016/j.mrgentox.2011.10.012 22094289

[B31] Liu Q. Lei Z. Huang A. Wu Q. Xie S. Awais I. (2017). Toxic metabolites, MAPK and Nrf2/Keap1 signaling pathways involved in oxidative toxicity in mice liver after chronic exposure to Mequindox. *Sci. Rep.* 7:41854 10.1038/srep41854 PMC5291092 28157180

[B32] Liu Q. Zhang J. Luo X. Ihsan A. Liu X. Dai M. (2016). Further investigations into the genotoxicity of quinoxaline-di-N-oxides and their primary metabolites. *Food Chem. Toxicol.* 93 145–157. 10.1016/j.fct.2016.04.029 27170491

[B33] Liu W. Gao X. Ma G. Yan L. Chen T. Li T. (2016). Correlation of genetic results with testicular histology, hormones and sperm retrieval in nonobstructive azoospermia patients with testis biopsy. *Andrologia* 49:e12705 10.1111/and.12705 27921326

[B34] Liu W. Y. Wang Z. B. Zhang L. C. Wei X. Li L. (2012). Tight junction in blood-brain barrier: an overview of structure, regulation, and regulator substances. *CNS Neurosci. Ther.* 18 609–615. 10.1111/j.1755-5949.2012.00340.x 22686334 PMC6493516

[B35] Liu Y. C. Si H. B. He L. M. Ding H. Z. Huang X. H. Chen J. X. (2010). Identification of mequindox and its metabolites by high performance liquid chromatography combined with ion trap-time of flight-mass spectrometry. *Chin. J. Anal. Chem.* 38 82–86. 10.3724/SP.J.1096.2010.00082

[B36] Liu Z. Y. Huang L. L. Chen D. M. Yuan Z. H. (2010). Metabolism of mequindox in liver microsomes of rats, chicken and pigs. *Rapid Commun. Mass Spectrom.* 24 909–918. 10.1002/rcm.4460 20196192

[B37] Liu Z. Y. Sun Z. L. (2013). The metabolism of carbadox, olaquindox, mequindox, quinocetone and cyadox: an overview. *Med. Chem.* 9 1017–1027. 10.2174/1573406411309080002 23521002

[B38] Lu Y. Luo B. Li J. Dai J. (2016). Perfluorooctanoic acid disrupts the blood-testis barrier and activates the TNFalpha/p38 MAPK signaling pathway in vivo and in vitro. *Arch. Toxicol.* 90 971–983. 10.1007/s00204-015-1492-y 25743374

[B39] NRC (2004). *The Development of Science based Guidelines for Laboratory Animal Care, Proceedings of the November 2003 International Workshop.* Washington, DC: National Academy Press. 20669462

[B40] OECD (2009). *Guideline for the Testing of Chemicals.* Paris: OECD.

[B41] Pena F. J. Plaza Davila M. Ball B. A. Squires E. L. Martin Muñoz P. Ortega Ferrusola C. (2015). The impact of reproductive technologies on stallion mitochondrial function. *Reprod. Domest. Anim.* 50 529–537. 10.1111/rda.12551 26031351

[B42] Pena F. J. Rodriguez Martinez H. Tapia J. A. Ortega Ferrusola C. Gonzalez Fernandez L. Macias Garcia B. (2009). Mitochondria in mammalian sperm physiology and pathology: a review. *Reprod. Domest. Anim.* 44 345–349. 10.1111/j.1439-0531.2008.01211.x 19144010

[B43] Plaza Davila M. Martin Munoz P. Tapia J. A. Ortega Ferrusola C. Balao da Silva C. C. Pena F. J. (2015). Inhibition of mitochondrial complex I leads to decreased motility and membrane integrity related to increased hydrogen peroxide and reduced ATP production, while the inhibition of glycolysis has less impact on sperm motility. *PLOS ONE* 10:e0138777 10.1371/journal.pone.0138777 PMC4583303 26407142

[B44] Poole J. S. Hadad C. M. Platz M. S. Fredin Z. P. Pickard L. Guerrero E. L. (2002). Photochemical electron transfer reactions of tirapazamine. *Photochem. Photobiol.* 75 339–345. 10.1562/0031-8655(2002)075<0339:PETROT>2.0.CO;2 12003122

[B45] Qiu L. Zhang X. Zhang X. Zhang Y. Gu J. Chen M. (2013). Sertoli cell is a potential target for perfluorooctane sulfonate-induced reproductive dysfunction in male mice. *Toxicol. Sci.* 135 229–240. 10.1093/toxsci/kft129 23761298

[B46] Ring J. Welliver C. Parenteau M. Markwell S. Brannigan R. E. Kohler T. S. (2017). The utility of sex hormone binding globulin in hypogonadism and infertile males. *J. Urol.* 197 1326–1331. 10.1016/j.juro.2017.01.018 28087298

[B47] Saunders P. T. (2003). Germ cell-somatic cell interactions during spermatogenesis. *Reproduction* 61 91–101. 14635929

[B48] Siu E. R. Mruk D. D. Porto C. S. Cheng C. Y. (2009). Cadmium-induced testicular injury. *Toxicol. Appl. Pharmacol.* 238 240–249. 10.1016/j.taap.2009.01.028 19236889 PMC2804910

[B49] Sohn J. Kim S. Koschorreck J. Kho Y. Choi K. (2016). Alteration of sex hormone levels and steroidogenic pathway by several low molecular weight phthalates and their metabolites in male zebrafish (*Danio rerio*) and/or human adrenal cell (H295R) line. *J. Hazard. Mater.* 320 45–54. 10.1016/j.jhazmat.2016.08.008 27513369

[B50] Vicente E. Perez-Silanes S. Lima L. M. Ancizu S. Burguete A. Solano B. (2009). Selective activity against Mycobacteriumtuberculosis of new quinoxaline 14-di-N-oxides. *Bioorg. Med. Chem.* 17 385–389. 10.1016/j.bmc.2008.10.086 19058970

[B51] Wang X. Fang G. J. Wang Y. L. Ihsan A. Huang L. L. Zhou W. (2011a). Two generation reproduction and teratogenicity studies of feeding cyadox in Wistar rats. *Food Chem. Toxicol.* 49 1068–1079. 10.1016/j.fct.2011.01.014 21266187

[B52] Wang X. Huang X. J. Ihsan A. Liu Z. Y. Huang L. L. Zhang H. H. (2011b). Metabolites and JAK/STAT pathway were involved in the liver and spleen damage in male Wistar rats fed with mequindox. *Toxicology* 280 126–134. 10.1016/j.tox.2010.12.001 21146578

[B53] Wang X. Bai Y. Cheng G. Ihsan A. Zhu F. Wang Y. (2016a). Genomic and proteomic analysis of the inhibition of synthesis and secretion of aldosterone hormone induced by quinocetone in NCI-H295R cells. *Toxicology* 350–352 1–14. 10.1016/j.tox.2016.03.005 27046791

[B54] Wang X. Martinez M. A. Cheng G. Liu Z. Huang L. Dai M. (2016b). The critical role of oxidative stress in the toxicity and metabolism of quinoxaline 14-di-N-oxides in vitro and in vivo. *Drug Metab. Rev.* 48 159–182. 10.1080/03602532.2016.1189560 27285897

[B55] Wang X. Wan D. Ihsan A. Liu Q. Cheng G. Li J. (2015). Mechanism of adrenocortical toxicity induced by quinocetone and its bidesoxy-quinocetone metabolite in porcine adrenocortical cells in vitro. *Food Chem. Toxicol.* 84 115–124. 10.1016/j.fct.2015.08.016 26296292

[B56] Wang X. Wu Q. Wan D. Liu Q. Chen D. Liu Z. (2015a). Fumonisins: oxidative stress-mediated toxicity and metabolism in vivo and in vitro. *Arch. Toxicol.* 90 81–101. 10.1007/s00204-00015-01604-00208 26419546

[B57] Wang X. Yang C. Ihsan A. Luo X. Guo P. Cheng G. (2016c). High risk of adrenal toxicity of N1-desoxy quinoxaline 14-dioxide derivatives and the protection of oligomeric proanthocyanidins (OPC) in the inhibition of the expression of aldosterone synthetase in H295R cells. *Toxicology* 34 1–16. 10.1016/j.tox.2016.01.005 26802905

[B58] Wang X. Yang P. P. Li J. Ihsan A. Liu Q. Y. Cheng G. Y. (2016d). Genotoxic risk of quinocetone and its possible mechanism in *in vitro* studies. *Toxicol. Res.* 5 446–460. 10.1039/C5TX00341E PMC6062406 30090359

[B59] Wang X. Zhang H. Huang L. Pan Y. Li J. Chen D. (2015b). Deoxidation rates play a critical role in DNA damage mediated by important synthetic drugs, quinoxaline 14-dioxides. *Chem. Res. Toxicol.* 28 470–481. 10.1021/tx5004326 25626015

[B60] Wu H. Li L. Shen J. Wang Y. Liu K. Zhang S. (2012). In vitro metabolism of cyadox in rat, chicken and swine using ultra-performance liquid chromatography quadrupole time-of-flight mass spectrometry. *J. Pharm. Biomed. Anal.* 67–68, 175–185. 10.1016/j.jpba.2012.04.004 22565170

[B61] Wu Y. Yu H. Wang Y. Huang L. Tao Y. Chen D. (2007). Development of a high-performance liquid chromatography method for the simultaneous quantification of quinoxaline-2-carboxylic acid and methyl-3-quinoxaline-2-carboxylic acid in animal tissues. *J. Chromatogr. A* 1146 1–7. 10.1016/j.chroma.2006.11.024 17335836

[B62] Yang W. Fu J. Xiao X. Yan H. Bao W. Wang D. (2013). Quinocetone triggers oxidative stress and induces cytotoxicity and genotoxicity in human peripheral lymphocytes of both genders. *J. Sci. Food Agric.* 93 1317–1325. 10.1002/jsfa.5891 23027643

[B63] Yang Y. Jiang L. She Y. Chen M. Li Q. Yang G. (2015). Olaquindox induces DNA damage via the lysosomal and mitochondrial pathway involving ROS production and p53 activation in HEK293 cells. *Environ. Toxicol. Pharmacol.* 40 792–799. 10.1016/j.etap.2015.09.008 26453893

[B64] Zhang J. Li Z. Qie M. Zheng R. Shetty J. Wang J. (2016). Sodium fluoride and sulfur dioxide affected male reproduction by disturbing blood-testis barrier in mice. *Food Chem. Toxicol.* 94 103–111. 10.1016/j.fct.2016.05.017 27237588

[B65] Zhang K. Ban M. Zhao Z. Zheng H. Wang X. Wang M. (2012). Cytotoxicity and genotoxicity of 14-bisdesoxyquinocetone, 3-methylquinoxaline-2-carboxylic acid (MQCA) in human hepatocytes. *Res. Vet. Sci.* 93 1393–1401. 10.1016/j.rvsc.2012.06.012 22840332

[B66] Zhang K. Wang X. Wang C. Zheng H. Li T. Xiao S. (2015). Investigation of quinocetone-induced mitochondrial damage and apoptosis in HepG2 cells and compared with its metabolites. *Environ. Toxicol. Pharmacol.* 39 555–567. 10.1016/j.etap.2015.01.017 25681706

[B67] Zhang K. Zheng W. Zheng H. Wang C. Wang M. Li T. (2014). Identification of oxidative stress and responsive genes of HepG2 cells exposed to quinocetone, and compared with its metabolites. *Cell Biol. Toxicol.* 30 313–329. 10.1007/s10565-014-9287-0 25223261

[B68] Zhao X. J. Huang C. Lei H. Nie X. Tang H. Wang Y. (2011). Dynamic metabolic response of mice to acute mequindox exposure. *J. Proteome Res.* 10 5183–5190. 10.1021/pr2006457 21905750

[B69] Zou J. Chen Q. Tang S. Jin X. Chen K. Zhang T. (2009). Olaquindox-induced genotoxicity and oxidative DNA damage in human hepatoma G2 (HepG2) cells. *Mutat. Res.* 676 27–33. 10.1016/j.mrgentox.2009.03.001 19486861

