# Supplementary figures and images for: Mechanisms of the Testis Toxicity Induced by Chronic Exposure to Mequindox
Source: Front Pharmacol. 2017 Sep 26;8:679. doi: 10.3389/fphar.2017.00679 (PMC5622959; doi:10.3389/fphar.2017.00679)

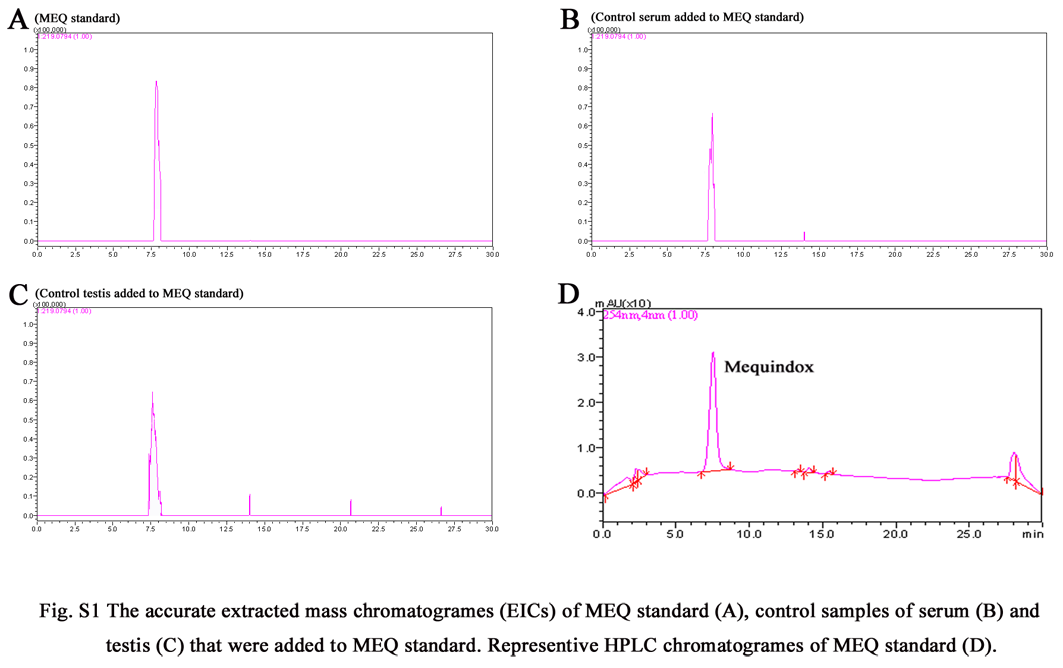

Supplement: Supplementary file 1 [file Image_1.TIF]
